# 

**Published:** 1884-03

**Authors:** 


					﻿VOLji
MARCH, 1884.
NO. 3.
THE
Homeopathic phy^iciaji,
A MONTHLY JOURNAL OF MEDICAL SCIENCE.
“Seek the Truths come whence it may, cost what it will."
ZE. T. LEM M. ID.,
EDITOR.
CONTENTS:
(See inside of Cover.)
PHILADELPHIA:
No. 2109 QHESTNUT STREET.
□ST 333 -W •sromc:
A. L. CHATTERTON, PUBLISHING COMPANY, Publisher’s Agents.
LONDON':
ALFRED HEATH & CO., Sole Agents fob Great Britain,
114 Ebury Street, Eaton Square.
Yearly Subscription, $2.50, invariably in advance.
Entered at the Philadelphia Post-Office as Second- Class Mail Matter.
				

